# Acute responsivity of the serotonergic system to S-citalopram and positive emotionality—the moderating role of the 5-HTTLPR

**DOI:** 10.3389/fnhum.2013.00486

**Published:** 2013-08-23

**Authors:** Catrin Wielpuetz, Yvonne Kuepper, Phillip Grant, Aisha J. L. Munk, Juergen Hennig

**Affiliations:** Personality Psychology and Individual Differences, Department of Psychology, Justus-Liebig-University GiessenGiessen, Germany

**Keywords:** S-citalopram, 5-HTTLPR, positive emotionality, serotonin, neuroendocrine challenge test

## Abstract

According to the idea that the central serotonergic system has a modulatory function on behavior and personality in general, we aimed to highlight its association to habitual positive emotionality. In a placebo-controlled double-blind and randomized cross-over neuroendocrine challenge design (*n* = 72 healthy males) we investigated the association of the central serotonergic responsivity, 5-HTTLPR-genotype as well as their combined effects on positive emotionality. Regression analyses revealed an involvement of the serotonergic system in positive emotionality. There was, however, no direct association between positive emotionality and cortisol responses to S-citalopram; rather 5-HTTLPR-genotype showed an association (*p* < 0.05). That is, positive emotionality scores increased with the number of s-alleles carried by the individuals. Most notable was the moderating role of 5-HTTLPR-genotype (*p* < 0.05) on the association between acute serotonergic responsivity and positive emotionality. Indeed, this association was only found in ss-homozygotes, in which the acute responsivity of the serotonergic system additionally seems to contribute to the level of positive emotionality (*r* = 0.70, *p* < 0.05). The findings correspond to previous research demonstrating that the 5-HTTLPR is not only involved in the negative-emotional aspects of behavior and temperament, but is associated, moreover, with positive affectivity—supporting the assumption of its valence-neutrality. In addition, our data are in line with the idea of possible influences of the 5-HTTLPR-genotype on early neuronal development. They also indicate the need for further studies in order to clearly elucidate the role of the serotonergic system and its subcomponents in the regulation of positive emotionality.

## Introduction

The central serotonergic system controls a wide range of processes (e.g., Lucki, 1998), wherein it is thought to have general non-specific inhibitory effects (Spoont, 1992). Most research has focused on the involvement of serotonin in psychopathology and negative affectivity, wherefore its potential contribution to positive affectivity has mostly been disregarded.

In his neurobiological theory Depue explicitly links the serotonergic system to these inhibitory processes on the neural and behavioral level, presuming it to function as a threshold-modulator facilitating the elicitation of avoidance- as well as approach-behavior in case of reduced functionality (Depue and Collins, 1999; Depue and Fu, 2011). It is, therefore, thought to be “valence-neutral” and should modulate negative as well as positive emotionality. On this background, the present study aims to further elucidate the role of interindividual variations in the functionality of the serotonergic system regarding the trait of positive emotionality—i.e., stable dispositions associated with frequent experiencing of positive affect, approach motivation and behavior, reflecting interindividual differences in underlying emotional response systems (Depue et al., 1994; Tellegen and Waller, 2008).

Eligible methods to assess the general activity of the central serotonergic system are neuroendocrine challenge paradigms, whereby the hormonal response to a serotonergic agonist indexes its net responsivity (Yatham and Steiner, 1993). Few serotonergic challenge-studies have reported data regarding traits of positive emotionality (i.e., extraversion), most of which have not shown a link between extraversion and serotonergic responsivity using different substances (e.g., Manuck et al., 1998; Flory et al., 2004; Brummett et al., 2008; Kuepper et al., 2010). Indeed, Reist et al. (1996) demonstrated a positive association of the cortisol response to paroxetin (indicating a diminished serotonergic responsivity) in healthy males. Further evidence stems from challenge-studies assessing mood over several consecutive days. While Zald and Depue (2001) showed a blunted prolactin response to fenfluramine to be associated with enhanced averaged negative as well as positive affect [i.e., the two independent dimensions of mood, as postulated by Watson et al. (1988)] in healthy males, Flory et al. (2004) only found an association with positive affect, albeit in opposite direction, in a considerably larger mixed-sex sample. Summarily, there are indications of an association of the responsivity of the serotonergic system with at least some aspects of positive emotionality; particularly when the affective-component is measured [i.e., indicated by moderately stable indicators of mood, measured over several weeks (Zald and Depue, 2001)].

Genetic variations could contribute to differences in serotonergic responsivity. One of the most intensively investigated serotonin-related polymorphisms is the 5-HTTLPR (serotonin transporter-gene-linked polymorphic region) within the promoter of the serotonin-transporter (5-HTT) gene (SCL6A4). The deletion/insertion of a repeat-element results in a short (s-allele) and a long allele (l-allele) (Lesch et al., 1996). Most research regarding the 5-HTTLPR and personality in healthy volunteers has stressed traits of negative emotionality (e.g., Munafo et al., 2009). Concerning positive emotionality, a meta-analysis by Munafo et al. (2003) concluded that there is no association with approach traits, which has been confirmed subsequently (Sen et al., 2004; Munafo et al., 2006; Kazantseva et al., 2008; Fox et al., 2009; Terracciano et al., 2010).

As temperamental traits are defined as emerging from a general sensitivity for trait-congruent emotional stimuli (e.g., Gray, 1982; Depue and Collins, 1999), studies assessing this responsiveness could additionally be consulted. Alongside an altered attention to unpleasant stimuli (Pergamin-Hight et al., 2012), some studies demonstrated that s-allele-carriers, unlike ll-homzygotes, also selectively attend to pleasant stimuli (Beevers et al., 2009, 2011; Fox et al., 2011). Neuroimaging studies underpin this association, showing heightened neuronal activation in response to negatively- (Munafo et al., 2008) as well as positively-valenced stimuli in s-allele-carriers (Canli et al., 2005; Herrmann et al., 2007; Klucken et al., 2012).

This greater responsiveness to both negative and positive stimuli in s-allele-carriers also seems to be evident regarding the impact of life events. Ss-homozygotes are disproportionally impaired more by negative life events (e.g., Uher and McGuffin, 2008; Karg et al., 2011), but also profit substantially more from the absence of negative life events—as reviewed by Belsky et al. (2009)—or a preponderance of positive life events (Taylor et al., 2006; Pluess et al., 2010; Kuepper et al., 2012).

These findings suggest that the s-allele of the 5-HTTLPR actually seems to be associated with a general “hypervigilance” (Homberg and Lesch, 2011) to emotional stimuli of negative as well as positive valence. Moreover, research on gene × environment (G × E) interactions could explain why there is no direct association between the 5-HTTLPR-genotype and positive emotionality, thus highlighting the moderating role of the 5-HTTLPR-genotype.

We investigated the association between two indicators of serotonergic functionality (i.e., acute neuroendocrine response to S-citalopram and the 5-HTTLPR-genotype) and habitual positive emotionality, expecting a moderating effect of 5-HTTLPR-genotype. This was based on findings regarding G × E interactions concerning psychological outcomes as well as first indications that serotonergic activity is especially influenced by environmental factors in s-allele-carriers (Bennett et al., 2002; Manuck et al., 2004). Thus, it is conceivable that the strength of an association between serotonergic functionality and positive emotionality varies depending on genotype. If the hypothesized effect is valid, a main effect of acute responsivity should be weak or absent, explaining previous inconclusive findings.

## Materials and methods

### Participants

Participants were recruited at the University of Giessen and via announcements in a local magazine; offering € 150 for participation. Seventy-two males were carefully selected on the basis of their 5-HTTLPR-genotype as well as a polymorphism in the TPH-2 gene (rs4570625; G-703T; for details see “Genotyping”) and if they met the following criteria: Caucasian ethnicity, age between 18 and 33, right-handedness, non-smoker for at least 1 year, no current or history of psychiatric or physical disorders (i.e., neurological or chronic diseases), no current use of any medication, no drug or alcohol abuse, coffee-consumption below 8 cups per day and a body mass index (BMI) between 19 and 25.4. The listed criteria were assessed via self-report. Additionally, subjects with a Beck Depression Inventory-score above 17 were excluded (German translation, Hautzinger et al., 1995), and an adapted version of the Mini-DIPS (Margraf, 1994) with the screening items in written format was administered. If necessary, the respective part of the structured interview was conducted.

Four participants had to be excluded after study-participation because of missing data (*N* = 1) or abnormal measurement results in cortisol samples (*N* = 3). Therefore, the final data analyses are based on 68 participants (mean age 24.2 ± 2.6 years).

Before participating in the study, participants were fully informed about the objective of the study, the administered substance, and possible side effects of S-citalopram, and written informed consent was obtained. They were further instructed how to behave on the day of the testing-sessions as well as on the evening before; essentially to refrain from alcohol-, medication- or drug-use, go to bed before 12 p.m. and have lunch before 1 p.m. on the day of the testing-session. The study protocol was approved by the ethics committee of the German Association of Psychology (DGPs).

### Personality

Positive Emotionality (PEM) was assessed through the brief form of the Multidimensional Personality Questionnaire (MPQ-BF, German translation: Angleitner et al., 1993). The MPQ-BF (Patrick et al., 2002) additionally measures Negative Emotionality and Constraint, with 155 items in total in a dichotomous format (true = 1, false = 0). The PEM-scale is comprised of four subscales: Wellbeing, Social Potency, Social Closeness, and Achievement. The total score is obtained by summing up the respective 36 items, yielding scores between 0 and 36.

### Procedure

Participants were given 10 mg S-citalopram (orally, Cipralex®, Lundbeck, Germany) during one testing-session and placebo during the other in a double-blind randomized crossover-design. We chose a dosage of 10 mg, because prior studies showed a lack of side effects as well as an equal proportion of responders and non-responders in the cortisol response—a desirable prerequisite to investigate interindividual differences in the responsivity of the serotonergic system (Kuepper et al., 2006).

The interval between the two sessions was exactly 14 days (20 days in only one individual) to ensure a sufficient wash-out period according to the pharmacokinetics of S-citalopram (Sogaard et al., 2005). The sessions started either at 14:50 h, 15:00 h, 15:10 h, or 15:20 h to enable the testing of four participants per day.

On arrival, participants were seated in an upright position in comfortable arm chairs for the whole testing-session. To prevent boredom, they were allowed to read provided neutral magazines. After a familiarization-period of 15 min the baseline saliva sample was obtained, directly followed by the administration of either verum or placebo. Further cortisol samples for the challenge paradigm were collected 50 min after drug intake and then in subsequent 30 min-intervals (samples 3–7). Before the second sample after 50 min, participants completed one of two questionnaire-sets during each testing-session.

Because of the long testing-interval and to prevent side effects, participants were offered 100 ml of water for the substance-intakes and, additionally, 50 ml after cortisol samples 2, 5, and 7 as well as 100 ml water and a soft pretzel after the third sample.

### Salivary cortisol and response measure

Saliva samples were collected with Salivettes® according to the standard-procedure (Sarstedt, Nuembrecht, Germany). After every test day saliva samples were centrifuged (10 min, 4000 × g) and frozen immediately at −30°C for later use. Concentrations of salivary cortisol levels were determined through use of a commercial enzyme immunoassay (IBL, Hamburg, Germany). All analyses were performed in duplicates using a fully automated analyzer (Adaltis, NexGen Four, Freiburg, Germany). All samples were analyzed within the same lot to avoid inter-assay-variation due to differences in different charges. The intra-assay-variation (CV) was lower than 5%.

**Figure 1:**
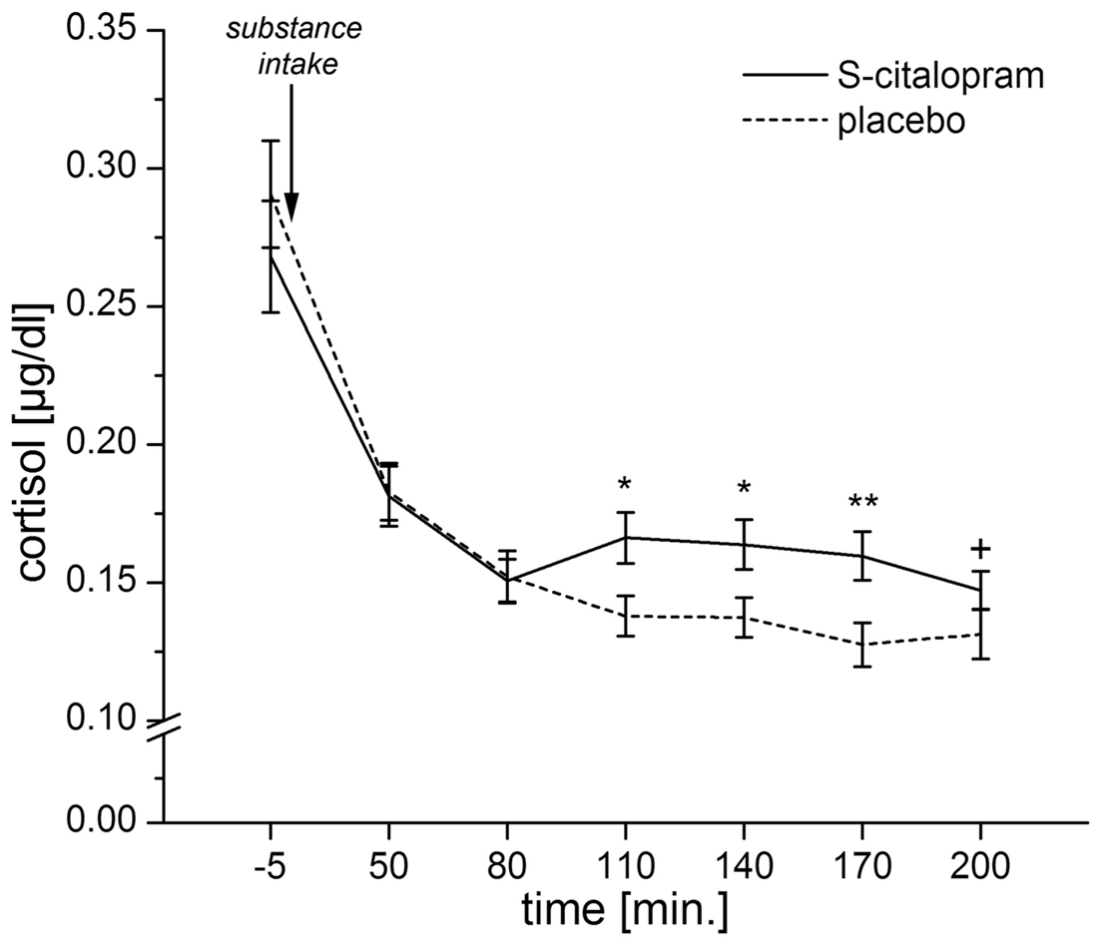
**Mean cortisol levels for both placebo and S-citalopram conditions for samples 1–7.** Error bars indicate the *SEM*. The area between the curves for S-citalopram and placebo for samples 3–7 (80–200 min after substance intake) is represented in the AUC-R. ANOVAs for repeated measures showed a significant interaction: substance × time point [*F*_(2.1, 144.1)_ = 3.71, *p* = 0.023, Greenhouse–Geisser-adjusted]. This analysis was based on *N* = 67 participants, as one participant had a missing cortisol-sample for the second time point. (^*^p < 0.01, ^**^p < 0.001, ^+^*p* = 0.066).

In order to have a single measure for the serotonergic responsivity, we calculated the placebo-corrected area under the response curve (AUC-R) for the cortisol response (Pruessner et al., 2003). A preliminary analysis of the cortisol responses showed significant differences between the cortisol responses under placebo and S-citalopram for samples 4–6 (*p*s ≤ 0.008) and a trend for sample 7 (*p* = 0.066). Therefore, areas under the curves (AUCs) were calculated for samples 3–7, using sample 3 as baseline. Finally, the placebo corrected AUC-R was determined by subtracting the AUC for placebo from the AUC for S-citalopram (Figure 1).

### Adverse side effects

To monitor possible side effects, participants' somatic symptoms were assessed using a visual analog scale (VAS)—ranging from 1 (no, not at all) to 100 (yes, completely)—with each saliva sample. The somatic symptom items comprised typical adverse side effects of S-citalopram, i.e., nausea, drowsiness, nasal congestion, dizziness, diarrhea, headache, mouth dryness and blurred vision. For statistical analysis regarding side effects, all somatic symptoms were summed up, separate for each time point (1–7). There was neither a significant effect of substance compared to placebo [*F*_(1, 67)_ = 1.68, *p* = 0.20] nor a significant interaction between substance and time point [*F*_(4.2, 278.3)_ = 0.76, *p* = 0.56]. Therefore, there were no adverse side effects of S-Citalopram, which was expected, due to the very low dosage of 10 mg (Kuepper et al., 2006).

### Genotyping

DNA was extracted from buccal cells using a standard commercial extraction kit (High Pure PCR Template Preparation Kit; Roche, Mannheim, Germany) in a MagNA Pure LC System (Roche). Genotyping for the 5-HTTLPR was performed in our lab as described previously by Alexander et al. (2009).

Participants were invited according to their genotype for 5-HTTLPR as well TPH-2 rs4570625, aiming at 4 equal cells. As part of another study, it was intended to investigate the joint influence of these two polymorphisms. Accordingly, participants were classified into s-allele-carriers or ll-homozygotes for the 5-HTTLPR (frequencies see Table 1) and T-allele-carriers vs. GG-homozygotes for the rs4570625.

**Table 1:** **Sample sizes, age, BMI, and AUC-R separated for 5-HTTLPR-genotype-groups (ss, sl, and ll)**.

	**ss**	**sl**	**ll**
*n*	10	22	36
age	24.40 ± 2.59	24.41 ± 2.93	24.06 ± 2.46
BMI (kg/m^2^)	23.33 ± 1.76	22.85 ± 1.87	22.53 ± 1.80
AUC-R	2.75 ± 7.78	3.04 ± 5.38	3.68 ± 8.19

*Means ± SDs for age, BMI, and AUC-R are reported*.

### Data analyses

To test for possible confounding factors (age, BMI), analyses of variance (ANOVA) or correlation analyses were conducted. Normal distributions of the PEM-scores and the AUC-R were verified through the Kolmogorov-Smirnov-test.

We tested for relevant predictors of PEM, using regression analyses, which enabled us to investigate potential mediator or moderator effects of 5-HTTLPR-genotype (more precisely, linear effects of an individual's number of s-alleles) and AUC-R (Baron and Kenny, 1986). In order to prevent problems of multicollinearity regarding the interaction term, AUC-R-values were standardized (AUC-R_*z*_) (Aiken and West, 1991).

First we conducted a regression analysis with only 5-HTTLPR-genotype entered to predict the AUC-R in order to test for the mediating effect.

In a second analysis, a hierarchical regression analysis (stepwise) was performed. Since neither PEM-scores nor 5-HTTLPR-genotype were associated with age and BMI (see results), these variables were not included. 5-HTTLPR-genotype (1 = ll, 2 = sl, 3 = ss, indicating an increasing number of 5-HTTLPR s-alleles), standardized AUC-R values (AUC-R_*z*_) and the interaction term 5-HTTLPR-genotype × AUC-R_*z*_ were entered consecutively. *Post-hoc* analyses for significant predictors were conducted using ANOVAs or correlation analyses. Results were considered significant with *p* < 0.05 (two-tailed).

## Results

Prior to the main analysis we tested whether 5-HTTLPR-genotype or AUC-R was associated with age or BMI. 5-HTTLPR-genotype-groups did not differ with respect to age [*F*_(2, 65)_ = 0.15, *p* = 0.86] or BMI [*F*_(2, 65)_ = 0.80, *p* = 0.46] (see Table 1). Likewise, the AUC-R was not significantly associated with age (*r* = 0.07, *p* = 0.55) or BMI (*r* = 0.11, *p* = 0.38).

PEM-scores (*M* = 31.11, *SD* = 8.23) as well as the AUC-R (*M* = 2.98, *SD* = 7.06) were distributed normally (PEM: *Z* = 0.81, *p* = 0.54; AUC-R: *Z* = 0.61, *p* = 0.76).

Regression analysis revealed no significant association between 5-HTTLPR-genotype (i.e., number of s-alleles) and AUC-R_*z*_ [β = 0.01; *R* = 0.04, *F*_(1, 66)_ = 0.13, *p* = 0.72, *R*^2^ = 0.002]. In order to exclude the possibility that differences between the genotype-groups were non-linear (and hence would not be detected by regression analyses), an ANOVA was performed. But likewise, there were no significant differences regarding AUC-R values between 5-HTTLPR-genotype-groups [*F*_(2, 65)_ = 0.07, *p* = 0.94; also see Table 1]. Consequently, it could be ruled out that the AUC-R functions as a mediator of a possible association of 5-HTTLPR-genotype and PEM.

A hierarchical regression analysis was conducted to examine whether markers of serotonergic system activity (i.e., 5-HTTLPR, AUC-R) predict PEM. Since age and BMI were not associated with either predictor and, additionally, were not associated with PEM-scores themselves (age: *r* = −0.10, *p* = 0.43; BMI: *r* = −0.01, *p* = 0.96), both variables were disregarded for further analyses. The first significant model [*R* = 0.24, *F*_(1, 66)_ = 4.2, *p* = 0.045; *R*^2^ = 0.059] showed only the 5-HTTLPR-genotype to be a significant predictor of PEM-scores (β = 0.24, *p* = 0.045), whereas the AUC-R_*Z*_ was non-significant and hence excluded (β = 0.23, *p* = 0.053). Inclusion of the interaction term lead to a second significant model [*R* = 0.35, *F*_(2, 65)_ = 4.5, *p* = 0.014; *R*^2^ = 0.122, Δ*R*^2^ = 0.063], rendering the interaction term 5-HTTLPR × AUC-R_*z*_ significant (β = 0.25, *p* = 0.034) and thereby minimally reducing the weight of the 5-HTTLPR-genotype (β = 0.23, *p* = 0.053). The AUC-R_*z*_ still remained excluded (β = 0.03, *p* = 0.90).

**Figure 2:**
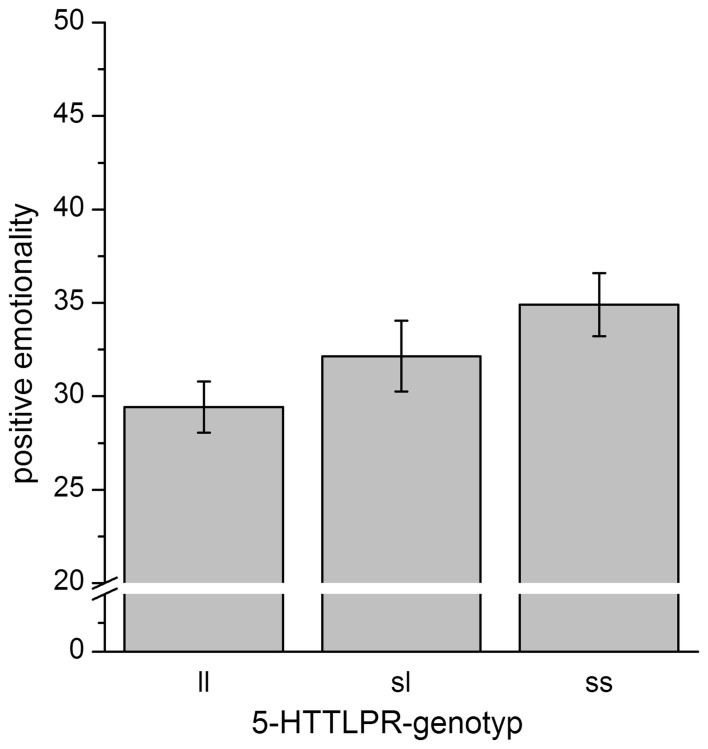
**Mean scores for positive emotionality separated for 5-HTTLPR-genotype.** Error bars indicate the *SEM*. While the ANOVA was insignificant, when contrasting only ss-homozygotes vs. ll-homozygotes, we detected a trend for significantly increased scores of positive emotionality in ss-homozygotes [*T*_(65)_ = 1.89, *p* = 0.063].

To further evaluate the results, *post-hoc* analyses were performed. Though the regression analysis revealed a linear association between the 5-HTTLPR-genotype and PEM-scores, differences between 5-HTTLPR-genotype-groups were not significant [*F*_(2, 65)_ = 2.1, *p* = 0.14], while the linear association can clearly be seen in the data (see Figure 2).

**Figure 3:**
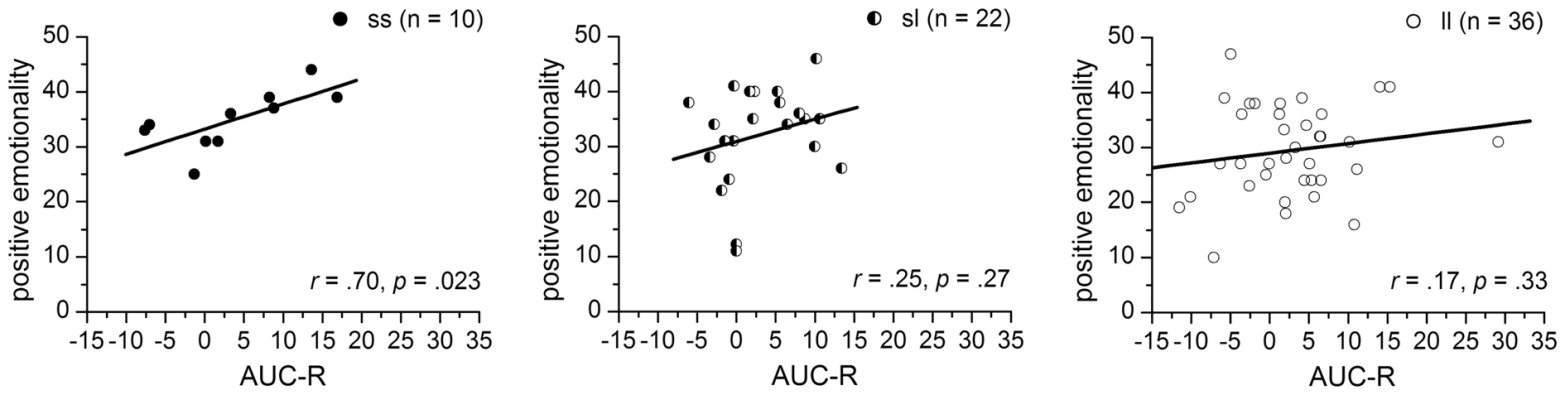
**Correlations between the AUC-R (raw data) and positive emotionality separated for 5-HTTLPR-genotype: ss, sl, and ll**.

Since the significant interaction term indicates 5-HTTLPR-genotype specific associations between the AUC-R_*Z*_ and PEM-scores, simple correlation analyses were performed separated for 5-HTTLPR-genotype-groups. We found a significantly positive correlation between AUC-R and PEM-scores for the ss-homozygotes (*r* = 0.70, *p* = 0.023) but no associations for either the sl-heterozygotes (*r* = 0.25, *p* = 0.27) or the ll-homozygotes (*r* = 0.17, *p* = 0.33) (see Figure 3).

Although the effect of the AUC-R was not significant, we repeated our analysis with keeping the AUC-R in the regression model. When entering both the AUC-R and the interaction term simultaneously, neither reached significance [AUC-R: β = 0.03, *p* = 0.91; AUC-R × 5-HTTLPR: β = 0.23, *p* = 0.39; *R* = 0.35, *F*_(3, 64)_ = 3.0], indicating only the 5-HTTLPR-genotype as a significant predictor of positive emotionality (β = 0.24, *p* = 0.045).

To get a clearer picture of the nature of associations depicted in our first regression model, however, we additionally analyzed the correlation between the AUC-R and positive emotionality only within the group of l-allele-carriers (i.e., sl and ll). Interestingly, we observed no association between the AUC-R and positive emotionality within this group (*r* = 0.18, *p* > 0.15). This clearly indicates that the aforementioned interaction term is based on the associations between AUC-R and positive emotionality within ss-homozygotes (*r* = 0.70, *p* = 0.023). We are convinced that this alternative strategy of data analysis turned out to confirm our previous regression analysis and the relevance of the interaction term.

## Discussion

Based on the assumption that the central serotonergic system has a non-specific modulatory function on behavior (Spoont, 1992; Depue and Collins, 1999) and not exclusively on avoidance behavior and negative affect, we aimed to further elucidate its contribution to positive emotionality.

We did not find a direct association between positive emotionality and the acute functionality of the serotonergic system but somewhat remarkably with the 5-HTTLPR-genotype. That is, an increasing number of s-alleles was associated with increasing positive emotionality. Most notable, however, was the hypothesized moderating role of the 5-HTTLPR-gentoype on the association between acute serotonergic activity and positive emotionality. Such an association was found exclusively in ss-homozygotes. Thereby, it seems that only in ss-homozygotes the acute serotonergic system activity contributes additionally to the degree of positive emotionality.

Furthermore, our design allowed us to investigate the influence of 5-HTTLPR-genotype on central serotonergic responsivity. *In vitro* studies indicate a functional impact of 5-HTTLPR-genotype, with a higher transcriptional efficiency of the 5-HTT-gene as well as increased serotonin reuptake for the l-allele compared to the s-allele (Lesch et al., 1996; Greenberg et al., 1999). Corresponding PET- and SPECT-studies, however, yielded contradictory results. Considering only those studies using [^11^C]DASB, the radioligand considered to be most appropriate (Willeit and Praschak-Rieder, 2010), some found an increased serotonin transporter binding in ll-homozygotes compared to s-allele-carriers (Praschak-Rieder et al., 2007; Reimold et al., 2007; Kalbitzer et al., 2009), while Murthy et al. (2010), using the largest sample, could not show a functional influence. Also Christian et al. (2009) did not find any association in non-human primates, likewise pointing to no unequivocal evidence for a functional influence *in vivo*.

Furthermore, our results are in line with those neuroendocrine challenge studies in healthy participants finding no or only weak evidence for functional consequences (Manuck et al., 2004; Smith et al., 2004; Wong et al., 2008). Only Whale et al. (2000) demonstrated a blunted prolactin response for ss-homozygotes. They, however, used clomipramine, which does not act selectively only on the serotonergic system (Tatsumi et al., 1997). Thus, evidence suggests that the 5-HTTLPR does not appear to have robust functional consequences *in vivo*.

In addition, certain aspects of our study design would have been advantageous to detect a putative association. With S-citalopram we used the most specific SSRI (Owens et al., 2001) currently available in a placebo-controlled design. This ideally ensures exclusive assessment of net responsivity of the serotonergic system. Furthermore, our sample was considerably larger than most of the studies cited and consisted of healthy males only. Thereby, we excluded confounding effects of varying estrogen levels across the menstrual cycle that exert influence on the hypothalamus as well as the serotonergic system (McEwen, 2001). In addition, the sample consisted of comparably young participants, which excluded an influence of age on the serotonin transporter binding potential as shown by Kalbitzer et al. (2009).

Nonetheless, we showed positive emotionality to increase with the number of s-alleles. This finding was remarkable, since nearly no study has demonstrated such an association when personality was measured (e.g., Lesch et al., 1996; Sen et al., 2004; Terracciano et al., 2010). This might be explained by two aspects. Firstly, while most of the respective studies (e.g., Lesch et al., 1996; Terracciano et al., 2010) used the NEO-PI-R (Costa and McCrae, 1992), we assessed positive emotionality rather than extraversion through the MPQ. The MPQ is considered to be an *emotional-temperament* inventory reflecting interindividual differences in the underlying emotional response systems (Tellegen, 1985; Patrick et al., 2002; Tellegen and Waller, 2008) of the traits of positive and negative emotionality that are conceptually parallel with Depue's postulated affective personality dimensions (Depue and Lenzenweger, 2001). This means that high scores in this scale for positive emotionality would indicate a heightened responsiveness to emotional stimuli. Specifically, such an increased reactivity for positive as well as negative emotional stimuli has been demonstrated in s-allele-carriers several times (e.g., Canli et al., 2005; Herrmann et al., 2007; Beevers et al., 2009). One could, insofar, speculate that the MPQ more closely depicts emotional responsiveness and reflects its biological underpinnings more adequately. Moreover, there is evidence that NEO extraversion and MPQ positive emotionality do not represent the same construct (Church, 1994).

A second explanation might be the highly selected sample. We carefully made sure to exclude participants with psychiatric disorders. When bearing G × E interactions in mind, it could be hypothesized that by excluding volunteers with higher depression symptoms we especially excluded s-allele-carriers with predominantly negative life events in favor of s-allele-carriers with a preponderance of positive life events and thus possibly higher positive emotionality. In other words, studies including participants with higher scores in neuroticism may be at risk of masking effects for positive emotionality. This hypothesis is further supported by Minelli et al. (2011), who demonstrated that associations between the 5-HTTLPR and anxiety-related traits only exist in samples heterogenous regarding the mental health status.

In conclusion, there is evidence that there is, indeed, a detectable albeit weak association between 5-HTTLPR-genotype and positive emotionality; whereby further research is needed to disentangle the specific effects of those factors mentioned above, which seem to influence the detection of this association. Hereof, life events appear to be an especially promising candidate.

The influence of 5-HTTLPR-genotype was not mediated by the acute responsivity of the serotonergic system. Instead, the 5-HTTLPR-genotype took on the role of a moderator; i.e., the functionality of the serotonergic system was positively linked to positive emotionality in ss-homozygotes only.

An elevated cortisol response can be interpreted as indexing a reduced overall activity of the serotonergic system. The putative reason for an elevated cortisol response is an upregulation or sensitization of postsynaptic receptors, caused by a habitually diminished serotonin release (Hennig et al., 2005). Within this line of reasoning, our results support Depue's theory, which postulates that a reduced serotonergic functionality lowers the threshold for response elicitation, which, in turn, should manifest in temperamental traits (Depue and Collins, 1999). Importantly, however, we demonstrated this effect in ss-homozygotes only.

At this point, one could only speculate why differences in the serotonergic responsivity are only linked to personality in ss-homozygotes. Despite the presumably lacking functional consequences of the 5-HTTLPR-genotype *in vivo*, evidence nevertheless suggests that it is associated with emotional processing and personality even in healthy participants.

To integrate these divergent findings, it seems plausible that the 5-HTTLPR-genotype exerts some of its effects primarily during the ontogeny of the central nervous system. Nordquist and Oreland (2010) as well as Whitaker-Azmitia (2005) have summarized findings regarding the neurotrophic effects of serotonin. They argue that an excess of serotonin during early neuronal development—which might be caused by the short variant of the 5-HTTLPR—could lead to morphological and functional changes, including circuits involved in emotional processing as well as in serotonergic neurons themselves, thereby likely leading to behavioral and emotional alterations in later life.

This reasoning fits to the observed alterations in neuronal circuits associated with emotional processing (e.g., Hariri et al., 2002; Canli et al., 2005; Heinz et al., 2005; Pezawas et al., 2005), which are likely to account for the hypervigilance in s-allele-carriers (Homberg and Lesch, 2011). Based on these considerations, one might interpret our data as indicating that acute variations in the functionality of the serotonergic system, indeed, only modulate positive emotionality in those subjects who are genetically predisposed. Underpinning this, there is evidence that 5-HTTLPR-genotype moderates the effects of, e.g., acute tryptophan depletion on mood and behavior (Marsh et al., 2006; Neumeister et al., 2006; Markus and Firk, 2009; Markus and De Raedt, 2011), pointing to a higher vulnerability to alterations in the serotonergic neurotransmission in ss-homozygotes.

The main reason for this study regarding the association of the serotonergic system and positive emotionality was Depue's postulate (Depue and Collins, 1999) of the serotonergic system as a general threshold modulator. One could further speculate on an essential contribution of the oxytocinergic system to positive emotionality, not least because Depue (Depue and Lenzenweger, 2001; Depue and Morrone-Strupinsky, 2005; Depue and Fu, 2011) also linked components of positive emotionality—namely affiliation—to the oxytocinergic system.

Indeed, there a several additional arguments that suggest that effects of oxytocin could have, at least partially, contributed to our results. Firstly, there is a co-localization of oxytocin-labeled cells and serotonin-transporter-labeled fibers in the paraventricular (PVN) and supraoptic nuclei (SON) of the hypothalamus (Emiliano et al., 2007). Secondly, oxytocin-release is influenced by acute serotonergic neurotransmission: exogenous serotonin as well as several serotonin-receptor agonists (primarily via 5-HT_1A_, 5-HT_2C_, and 5-HT_4_ receptors) lead to an increase in plasma oxytocin levels (Jorgensen et al., 2003). Importantly, the acute administration of citalopram also increases the concentration of oxytocin in the plasma (Uvnas-Moberg et al., 1999) as well as levels of oxytocin-mRNA within the magnocellular regions of the PVN (Hesketh et al., 2005). Regarding a possible influence on the cortisol-release to S-citalopram in our study, there is evidence that central oxytocin-release leads to attenuated endocrine responses to acute stress; i.e., glucocorticoids and ACTH, as was shown in studies administering oxytocin directly (Windle et al., 1997; Heinrichs et al., 2003; Parker et al., 2005; Ditzen et al., 2009).

The interaction of both systems, therefore, could have contributed to the association of positive emotionality with the cortisol-response to S-citalopram to some degrees (also, if in ss-homozygotes only). The exact consequences of the influence of oxytocin are, however, hard to specify (because of a contribution of levels of both neurotransmitters as well as various involved receptors). One could only speculate, whether oxytocinergic influences also contribute to our association of 5-HTTLPR-genotype with positive emotionality, which cannot be ruled out; not least due to additional neurodevelopmental effects of serotonin on the oxytocinergic system (Whitaker-Azmitia, 2005). Future research is needed to allow further conclusions in this respect (e.g., measures of plasma oxytocin-levels in a neuroendocrine challenge-paradigm).

Another point to keep in mind when interpreting our data, is that in light of the postulated general hypervigilance associated with the 5-HTTLPR s-allele (Homberg and Lesch, 2011), as well as Depue's understanding of the serotonergic system as a threshold modulator for both, approach and avoidance (e.g., Depue and Collins, 1999), one would expect similar results for the personality traits of negative emotionality. Therefore, we reanalyzed our data for negative emotionality (also assessed using the MPQ). Importantly, positive and negative emotionality are assumed to be two independent dimensions (e.g., Tellegen and Waller, 2008), which is supported by our data (*r* = −0.14, *p* = 0.24). The regression analysis did not show the 5-HTTLPR-genotype, the AUC-R or the interaction term to be associated with negative emotionality (for all βs, *p*s > 0.50). Though somewhat surprising, this result clarifies that the association of the serotonergic predictors of positive emotionality do not simply reflect an association with negative emotionality, but rather a distinct association with positive emotionality. Nevertheless, based on the above mentioned assumptions, one would rather have expected associations to both, positive and negative emotionality. Returning to our foregoing explanation for the results regarding positive emotionality and the 5-HTTLPR-genotype (i.e., the highly selected sample), this also seems to explain this possible lack of association. Following this explanation, we potentially excluded especially 5-HTTLPR s-allele-carriers with a high incidence of negative life events that display higher scores in neuroticism (e.g., Kuepper et al., 2006; Pluess et al., 2010) and more depression symptoms (e.g., Caspi et al., 2003; Taylor et al., 2006). Likewise, this is in accordance with the results by Minelli et al. (2011), showing that the 5-HTTLPR-genotype is only associated with anxiety-related traits in heterogenous samples.

Certain limitations of our study must be kept in mind: as far as molecular genetic studies are concerned, our sample was relatively small, and we only tested males. Replications are therefore clearly needed, especially in females. Furthermore, our reasoning concerning the underlying mechanisms is speculative, and future research should shed light on factors that (a) foster the association between the 5-HTTLPR and positive emotionality and (b) contribute to the acute serotonergic functionality that in turn could also shape personality traits. Reasonable candidates would be life events or other genetic variations.

Initial evidence for an influence of living conditions stems from a study by Manuck et al. (2004). They demonstrated that the association between the prolactin response to fenfluramine and the socio-economic status is moderated by 5-HTTLPR-genotype. This finding is further supported by Bennett et al. (2002), who found that the influence of early rearing conditions on the concentration of the serotonin metabolite 5-hydroxyindolacetic acid (5-HIAA) in the cerebrospinal fluid is moderated by the rh5-HTTLPR-genotype in rhesus macaques.

Beyond this, further research should investigate the stability of the responsivity of the serotonergic system, which seems to be influenced by life events.

To our knowledge, this was the first study to specifically investigate the combined effects of both stable genetic variations and the acute responsivity of the serotonergic system on variations in positive emotionality. According to Depue, a diminished serotonergic functionality should ultimately amplify both positive and negative emotionality (Depue and Collins, 1999). Our results partly support this assumption in that ss-homozygotes manifest the highest positive emotionality. Furthermore, at least in ss-homozygotes, a reduced serotonergic functionality is, in turn, associated with higher positive emotionality. The results highlight the need to focus not only on negative affectivity and related processes but also on positive affectivity to fully understand the role of the serotonergic system.

### Conflict of interest statement

The authors declare that the research was conducted in the absence of any commercial or financial relationships that could be construed as a potential conflict of interest.
